# A U-Shaped Relationship between the Prevalence of Frailty and Body Mass Index in Community-Dwelling Japanese Older Adults: The Kyoto–Kameoka Study

**DOI:** 10.3390/jcm9051367

**Published:** 2020-05-06

**Authors:** Daiki Watanabe, Tsukasa Yoshida, Yuya Watanabe, Yosuke Yamada, Misaka Kimura

**Affiliations:** 1 National Institute of Health and Nutrition, National Institutes of Biomedical Innovation, Health and Nutrition, Tokyo 162-8636, Japan; yoshida@nibiohn.go.jp (T.Y.); yuwatana@mail.doshisha.ac.jp (Y.W.); yamaday@nibiohn.go.jp (Y.Y.); 2 Institute for Active Health, Kyoto University of Advanced Science, Kyoto 621-8555, Japan; kimura.misaka@kuas.ac.jp; 3 Laboratory of Applied Health Sciences, Kyoto Prefectural University of Medicine, Kyoto 602-8566, Japan; 4 Senior Citizen’s Welfare Section, Kameoka City Government, Kyoto 621-8501, Japan; 5 Faculty of Health and Sports Science, Doshisha University, Kyoto 610-0394, Japan

**Keywords:** body mass index, frailty, older adults, cross-sectional study

## Abstract

The relationship between body mass index (BMI) and frailty remains unclear. Using two validated frailty assessment tools, this study aimed to investigate the relationship between the prevalence of frailty and BMI in Japanese older adults. This cross-sectional study used baseline data of 7191 individuals aged ≥65 years, living in Kameoka City, Kyoto, Japan. The BMI was calculated based on self-reported height and body weight, and classified into six categories. Frailty was defined using two validated assessment tools, the Fried phenotype (FP) model and Kihon Checklist (KCL). We evaluated the relationship between frailty and BMI using a multivariate restricted cubic spline logistic regression. The prevalence of frailty defined using the FP model was 25.3%, 19.6%, 14.3%, 12.4%, 12.6%, and 19.4% for each BMI category of <18.5, 18.5–19.9, 20.0–22.4, 22.5–24.9, 25.0–27.4, and ≥27.5 kg/m^2^, respectively. The spline model showed a significant U-shaped relationship between BMI and the prevalence of frailty defined using both, KCL and FP models. This study found that the BMI range corresponding to lowest prevalence of frailty defined using both tools was 21.4–25.7 kg/m^2^. Thus, a healthy BMI may reduce the prevalence of frailty, and the risk of frailty needs to be evaluated in individuals who are underweight or overweight.

## 1. Introduction

Frailty is a condition, in which the function of multiple physiological systems is impaired due to a decrease in homeostasis in response to stress [1,2]. It is a geriatric syndrome that increases in prevalence with age [3], and is a multifactorial syndrome influenced by physiological, nutritional, social, and cognitive factors [4]. In older individuals, frailty is associated with adverse events, such as the risk of death [5,6,7] and disability [5,6,8]. Thus, to prolong the healthy lifespan of older people and reduce the burden of medical and caregiving expenses, it is necessary to reduce the number of people with frailty [8] and to establish a sustainable, comprehensive, and effective public health program to prevent frailty.

The body mass index (BMI) is a convenient scale for evaluating thinness and fatness. In a meta-analysis including data from not only Japanese people [9] but also those from around the world [10], the BMI associated with the lowest risk of death increased with age. However, frailty individuals who, based on BMI, are underweight (<18.5 kg/m^2^), of normal weight (18.5–24.9 kg/m^2^), or obese (≥30.0 kg/m^2^) have a higher risk of death than those who are not frailty [11]. Elucidating the relationship between BMI and the prevalence of frailty is therefore important for determining the prognosis of older individuals.

A cross-sectional study on several British subjects showed that BMI has a U-shaped relationship with the prevalence of frailty; it also showed that the BMI ranges associated with the lowest prevalence of frailty are 18.5–24.9 [12,13] and 25–29.9 kg/m^2^ [14]. Although the optimum BMI range in these studies varies, their findings suggest that it is important to maintain a healthy BMI to prevent frailty. However, the mean BMI differs between the Japanese and British people [15], and it is difficult to extrapolate the results of previous studies to account for the Japanese people. To our knowledge, there is no current evidence about the relationship between BMI and the prevalence of frailty in Japanese older people. This study aimed to investigate the relationship between the prevalence of frailty and the BMI in community-dwelling Japanese older adults, with the use of two validated frailty assessment tools.

## 2. Materials and Methods

### 2.1. Study Population

This cross-sectional study utilized data from the Kyoto–Kameoka Study, a cohort study of individuals aged ≥65 years who lived in Kameoka City, Kyoto Prefecture, Japan. On 29 July 2011, the Kyoto–Kameoka Study conducted a comprehensive baseline survey of needs in the sphere of daily life, which included the Fried phenotype (FP) model based Frailty Screening Index (FSI) and Kihon Checklist (KCL), in 18,231 individuals; the details are explained elsewhere [4,16,17,18]. An additional survey on health and nutritional status was also administered on 14 February 2012, and valid responses were obtained from 8319 participants. In both surveys, the questionnaires were recovered by post, and informed consent was obtained from participants who returned the questionnaires. Health-related information, including medical history, socioeconomic status, smoking habit, alcohol consumption, and physical activity, was extracted from both surveys.

Among these participants included in the study from baseline (*n* = 8319), we excluded those with incomplete responses to the FP model [8] and KCL [6,19], which evaluates frailty (*n* = 1096), those with missing BMI data (*n* = 11), and those with implausible BMI data (BMI of <14 or ≥40 kg/m^2^) (*n* = 21) [20] from the analysis. Ultimately, we included 7191 participants in this study (whole cohort).

This study was approved by the ethics review boards of the Kyoto Prefectural University of Medicine (No. RBMR-E-363) and the National Institute of Health and Nutrition (No. NIHN187-3). The study was carried out in accordance with the principles of the Declaration of Helsinki.

### 2.2. BMI and Other Variables

The BMI was calculated by dividing self-reported body weight (kg) by the square of height (m). The Kyoto–Kameoka Study asked participants about their height and body weight in both, the baseline and additional surveys. We used the BMI data of the baseline survey, which was the same survey used for the FP model and KCL investigation. However, variables based on self-reported data may be affected by systematic errors from recall bias in older people. In March and April 2012, the Kyoto–Kameoka Study conducted a physical examination by recruiting 1379 participants through cluster sampling (which randomly selected 10 districts from a total of 21 districts within Kameoka City) and measuring their height and body weight [17,18]. With the participant barefooted and wearing light clothing, the height and body weight were measured to the nearest 0.1 cm and 0.1 kg, respectively, using a digital height and weight scale (DST-210S; Muratec-KDS Corp., Kyoto, Japan). In participants whose height and body weight were obtained from both, the baseline and additional surveys (*n* = 1169), we evaluated the validity of the self-reported height, body weight, and BMI by comparing them with the measurements (subcohort). Such comparison of variables from the two surveys and from the physical examination, we evaluated the validity and reproducibility of the results. We classified the participants into the following BMI categories, as described in a previous study [10]: <18.5, 18.5–19.9, 20.0–22.4, 22.5–24.9, 25.0–27.4, and ≥27.5 kg/m^2^. We considered ≥27.5 kg/m^2^ as the highest BMI range because our study population had very few people with obesity (BMI ≥30 kg/m^2^).

### 2.3. Definition of Frailty

Frailty was evaluated using the self-administered KCL [4,6,16], which comprised 25 questions, and FP model based self-administered FSI [8], which consisted of 5 questions. Both tools had already been validated. The FP model is evaluated primarily in terms of physical aspects [8,13,19], whereas defined on the basis of the KCL is evaluated in multifaceted terms, taking account of social and cognitive aspects as well as physical factors [6,14,19]. Frailty, when evaluated using the FP model, was defined as a score of ≥3 out of 5 points [8]. According to a prospective cohort study, the FP model can also predict the risk of long-term care (LTC) after 2 years [8]. The KCL has seven subdomains that relate movements to instrumental activities of daily living (IADL), physical function, nutritional status, oral function, social status, cognitive status, and depression. The KCL has a score range of 0 (no frailty) to 25 points (high frailty), and frailty was defined as a score of ≥7 out of 25 points [4,16,19]. A prospective study has shown that the KCL can predict the risk of death or long-term care (LTC) after 3 years [6]. The cutoff scores of the seven KCL subdomains were evaluated according to a previous study [21]. Among the seven subdomains, we excluded the nutritional domain, as it was also evaluated when evaluating BMI; thus, we only evaluated the relationship between BMI and the other six subdomains.

### 2.4. Statistical Analysis

Regarding participant characteristics, continuous variables are shown as means and standard deviations, while categorical variables are shown as numbers of people and percentages. Missing values of covariates were supplemented from five data sets created by the multiple imputation method through multivariate imputation by chained equation, using R statistical software [22]. All missing values were assumed as missing at random.

Height, body weight, and BMI are expressed as means and standard deviations. To evaluate the validity and reproducibility of self-reported height, body weight, and BMI from the baseline survey, we used a *t-*test and an intraclass correlation coefficient, which were compatible with the actual measurements and data from the additional survey.

The prevalence of frailty for each BMI group is shown as numbers of cases and percentages. The relationship between BMI and the prevalence of frailty was evaluated by multivariate logistic regression analysis using the following models: Model 1, which was adjusted for age, sex, and region, and Model 2, which was adjusted for smoking habit, alcohol consumption, educational level, number of drugs taken, family composition, economic status, physical activity, presence/absence of dentures, and history of hypertension, stroke, heart disease, diabetes, and dyslipidemia in addition to age, sex, and region. These models were decided with reference to covariates used in previous studies, which examined the association between frailty and protein intake [4]. The results of these analyses are shown as odds ratios (ORs) and 95% confidence intervals (CIs). OR was calculated using the BMI range 22.5–24.9 kg/m^2^ as reference, based on previous research [23]. Additionally, the results were stratified according to age (<75 or ≥75 years) and sex.

We evaluated the relationship curve between the BMI and the prevalence of frailty, as defined by the FP model and KCL, using a restricted cubic spline model based on six points (mean BMI of each category) according to the BMI categories described previously. Owing to sparse data, we truncated analysis at 16.0 kg/m^2^ (1% of distribution) and 31.2 kg/m^2^ (99% of distribution) [24]. We calculated the ORs for prevalence of frailty associated with BMI, using the BMI of 23.0 kg/m^2^ as reference in the restricted cubic spline model. We also examined the relationship between the KCL subdomains and BMI in a similar manner.

Statistical significance was set to be <5% on both sides. All statistical analyses were performed using Stata/MP version 15.0 (StataCorp LP, College Station, TX, USA) and/or R software version 3.4.3 (R Core Team, Vienna, Austria).

## 3. Results

Table 1 shows the characteristics of the participants in the whole cohort and subcohort. Participants from the subcohort, whose height and body weight were measured, tended to have fewer current smokers and more people with a high level of physical activity than participants from the whole cohort; however, the differences were not significant.

Table 2 presents the validity and reproducibility of the self-reported height, body weight, and BMI. Differences were found between the mean values of measured height, body weight, and BMI and the mean values of the same parameters that were self-reported; however, the differences were also not significant. However, these variables showed a strong positive correlation with one another (self-reported vs. measured). Furthermore, height, body weight, and BMI, which were self-reported twice were highly reproducible. The relationships were the same even after the results were stratified according to sex and age.

Table 3 shows the characteristics of participants according to BMI. A larger number of people with higher BMI tended to be men with a history of hypertension, heart disease, diabetes, or dyslipidemia and tended to be younger. These individuals also tended to consist of fewer smokers, who had a low rate of use of dentures.

Table 4 shows the relationship between the prevalence of frailty and BMI on multivariate analysis. The prevalence of frailty defined using the FP model and KCL were 15.2% and 36.6%, respectively. The prevalence of frailty defined by the FP model and KCL were 25.3% and 55.5%, 19.6% and 37.7%, 14.3% and 34.2%, 12.4% and 32.6%, 12.6% and 34.3%, and 19.4% and 49.2% for each BMI category of <18.5, 18.5–19.9, 20.0–22.4, 22.5–24.9, 25.0–27.4, and ≥27.5 kg/m^2^, respectively, thereby showing a U-shaped relationship. We found a positive relationship between the prevalence of frailty defined by the FP model and both, low BMI and high BMI even after adjusting for baseline confounders when using 22.5–24.9 kg/m^2^ as the reference (BMI of <18.5 kg/m^2^: OR, 2.04; 95% CI, 1.58–2.63; BMI of 18.5–19.9 kg/m^2^: OR, 1.69; 95% CI, 1.33–2.14; BMI of 20.0–22.4 kg/m^2^: OR, 1.16; 95% CI, 0.96–1.41; BMI of 22.5–24.9 kg/m^2^: reference OR and CI; BMI of 25.0–27.4 kg/m^2^: OR, 1.00; 95% CI, 0.78–1.27; BMI of ≥27.5 kg/m^2^: OR, 1.54; 95% CI, 1.15–2.07). Frailty, when evaluated using the KCL, these results were also the same. Furthermore, these relationships were also the same even after the results were stratified according to sex and age (Tables S1 and S2).

**Figure 1:**
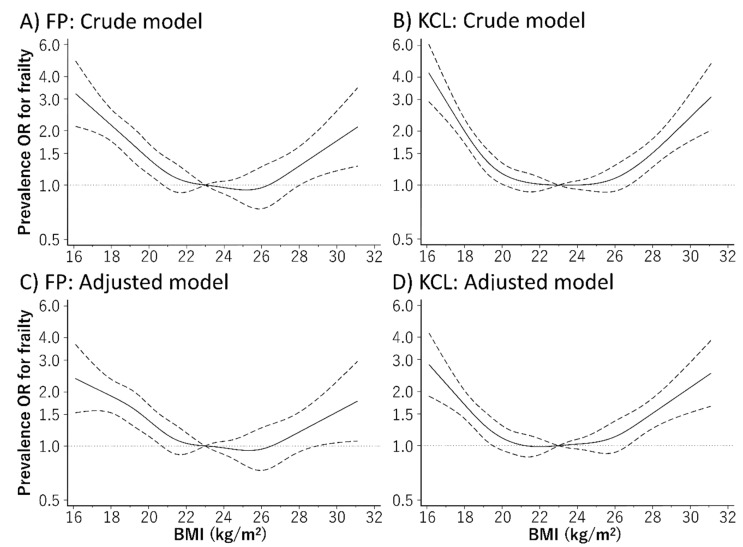
Relationship between body mass index (BMI) and frailty defined based on (**A**,**C**) Fried phenotype (FP) model and (**B**,**D**) the Kihon Checklist (KCL), using a restricted cubic spline logistic regression model. Frailty according to the KCL was defined as a score ≥7 out of 25 points, whereas frailty according to the FP model based self-administered frailty screening index (FSI) was defined as a score of ≥3 out of 5 points. Solid lines represent odds ratios (ORs), and dashed lines represent 95% confidence intervals (CIs). The OR base on a BMI of 23.0 kg/m^2^ as reference was calculated. This analysis included 7044 participants. We estimated that *p* < 0.05 when the 95% CI of the OR did not exceed 1.00, and *p* ≥ 0.05 when the 95% CI of the OR exceeded 1.00. The analysis was adjusted for age, sex, region, smoking habit, alcohol consumption, education history, number of drugs taken, family composition, economic status, physical activity, presence/absence of dentures, and history of hypertension, stroke, heart disease, diabetes, and dyslipidemia.

**Figure 2:**
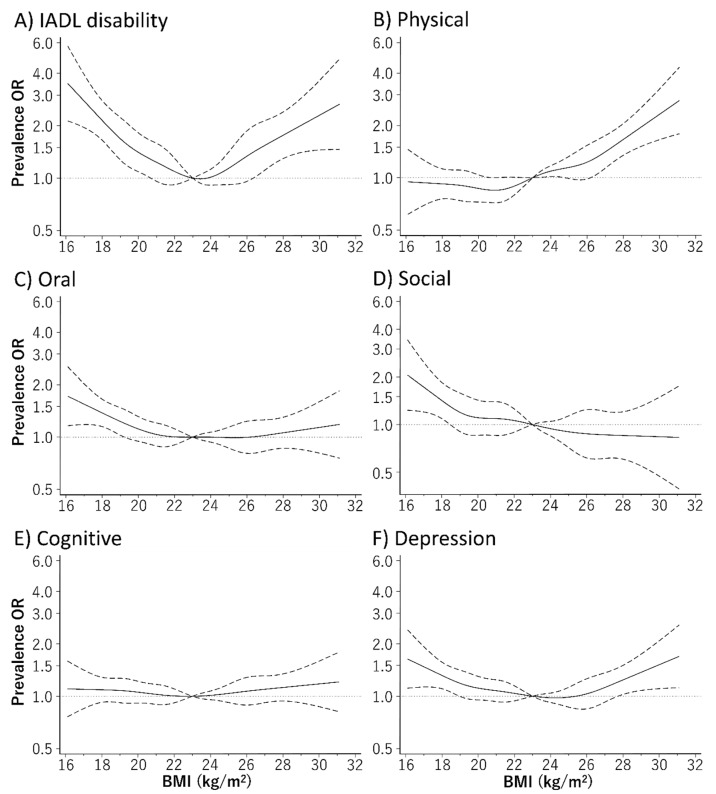
Relationship between body mass index (BMI) and prevalence rate of frailty based on the subdomains of the Kihon Checklist (KCL), using a restricted cubic spline logistic regression model. The prevalence rates of the six subdomains evaluated using the KCL were (**A**) 9.2% for instrumental activities of daily living (IADL) disability, (**B**) 22.7% for physical frailty, (**C**) 24.0% for oral frailty, (**D**) 8.3% for social frailty, (**E**) 36.5% for cognitive frailty, and (**F**) 31.6% for depression. Solid lines represent odds ratios (ORs), and dashed lines represent 95% confidence intervals (CIs). The OR based on a BMI of 23.0 kg/m^2^ as reference was calculated. We estimated that *p* < 0.05 when the 95% CI of the OR did not exceed 1.00, and *p* ≥ 0.05 when the 95% CI of the OR exceeded 1.00. The analysis was adjusted for age, sex, region, smoking habit, alcohol consumption, education history, number of drugs taken, family composition, economic status, physical activity, presence/absence of dentures, and history of hypertension, stroke, heart disease, diabetes, and dyslipidemia.

Figure 1 depicts the relationship curve between BMI and the prevalence of frailty using the restricted cubic spline model. Even after adjusting for baseline confounders, the relationship between BMI and the prevalence of frailty defined using the FP model and KCL showed a U-shaped curve. The BMI range with the lowest prevalence rate of frailty defined using the FP model and KCL were 24.7–25.7 kg/m^2^ and 21.4–22.8 kg/m^2^, respectively. The prevalence of IADL disability and depression evaluated using the KCL subdomains also showed a U-shaped relationship with the BMI (Figure 2). Furthermore, a low BMI was associated with a high prevalence of oral and social frailty, while a high BMI showed a positive relationship with the prevalence of physical frailty.

## 4. Discussion

In this study, we investigated the relationship between BMI and the prevalence of frailty using data from a population-based cohort study of older individuals. Even after adjusting for confounders, the BMI showed a U-shaped relationship with the prevalence of frailty as defined by both, the FP model and KCL, which are validated frailty assessment tools. The BMI range, for which the prevalence of frailty was the lowest, was 21.4–25.7 kg/m^2^. To our knowledge, this is the first study to show a positive relationship between the prevalence of frailty and BMI in Japanese people who are underweight (BMI < 18.5 kg/m^2^) and overweight (BMI ≥27.5 kg/m^2^). These findings suggest the need to evaluate the risk of frailty, not only in underweight people but also in overweight people.

This study showed that a low BMI was associated with a high prevalence of frailty. Several guidelines use BMI < 18.5 kg/m^2^ as a diagnostic marker for malnutrition [25,26], which may be a risk factor for frailty, because maintaining a healthy BMI in an older person is important for maintaining a healthy skeletal muscle mass and nutrition status [25,26,27]. Furthermore, our findings showed that a low BMI was associated with a high prevalence of oral frailty and social frailty, evaluated using the KCL subdomains. It has been reported that energy intake in older people is associated with eating together [28] and poor oral conditions [27,29]. Although the relationship between a low BMI and the prevalence of oral frailty and social frailty may be causally reversed, our results could provide important insight into risk factors for frailty, that need more careful attention in individuals with a low BMI. Furthermore, to prevent frailty, it would be necessary to establish target values for indicators, including BMI, which can evaluate energy balance. Although the Dietary Reference Intakes for Japanese (2020) establishes a personal target BMI range of 21.5–24.9 kg/m^2^ by considering the need to prevent both, frailty and onset of lifestyle-related diseases in older people [30], there is no clear basis for this target to prevent frailty. Our results indicate that the BMI range corresponding to the lowest prevalence rate of frailty was 21.4–25.7 kg/m^2^, which would support the BMI target value in the above guideline. Since a low BMI is a major risk factor for frailty in older people, it is therefore important to set a target BMI by considering individual characteristics and factors of lifestyle-related diseases.

Our results also showed that the prevalence of frailty is high even for individuals with a high BMI. Several previous studies have shown that the relationship between BMI and the prevalence of frailty is U-shaped [12,13,14,31]. A high BMI reflects a routinely high level of energy intake, rather than the amount of energy needed [32]. In the Wisconsin Primate Calorie Restriction study, a decrease in physical activity level was prevented to a greater extent in calorie-restricted rhesus monkeys than in rhesus monkeys fed on an ad libitum basis, and cut the metabolic cost associated with old age [33]. In addition, caloric restriction is related to a low incidence of frailty as defined by unintentional weight loss, significant reduction in spontaneous physical activity, and decreased kinetic energy efficiency [34]. Our results indicate that the prevalence of physical frailty defined using the KCL subdomain was high in overweight individuals, and these previous studies support our results. Therefore, to prevent frailty, it would be important to control body weight and to evaluate the risk of frailty in overweight people.

The strength of this study is that we were able to show the relationship between BMI and the prevalence of frailty defined using two validated assessment tools. Furthermore, as we have confirmed the validity of self-reported BMI by comparing it with measured BMI, a misclassification of self-reported BMI would be unlikely. Therefore, our results showed a strong relationship between variables and are therefore highly generalizable. However, our study has some methodological limitations. First, it has a cross-sectional design. Therefore, we cannot account for temporal and direct causal relationships between BMI and the prevalence of frailty. Second, our study included participants with a history of diabetes, dyslipidemia, heart disease, and stroke. These limitations may prevent generalization of the results. However, our results were similar after excluding participants with these diseases. Finally, we were unable to completely eliminate systematic errors from self-reporting. Thus, self-reported data such as education, income, smoking habit, and medical history may be affected by recall bias. However, as we used multivariate analysis to adjust for factors such as social and economic status, which are known to be associated with the results of frailty and BMI, we believe we were able to minimize the effect of confounding factors.

Hanlon et al. reported that the prevalence of frailty defined using the FP model (weight loss, exhaustion, grip strength, low physical activity, and slow walking pace) in 493,737 British individuals aged 37–73 years showed a difference of only around 2% between individuals aged 37–64 years and individuals aged ≥65 years [13]. Furthermore, the relationship between BMI and the prevalence of frailty in this population was U-shaped [13]. Since April 2008, Japan has been implementing the Specific Health Checkups and Specific Health Guidance and has since been evaluating the risk of metabolic syndrome in individuals aged 40–74 years [35]. Middle-aged and older individuals with obesity and metabolic syndrome may already be experiencing frailty; hence, adding items to current tools used for evaluating frailty among middle-aged and older individuals and evaluating metabolic syndrome and frailty at the same time are important. However, the concept of frailty and the use of screening methods in middle-aged people need to be applied with care to ensure that the results are similar to those in older people [13]. Furthermore, although appropriate diet [4] and physical activity [31] are crucial for preventing frailty, it is highly unlikely that a single method of intervention will apply to all individuals with frailty; thus, the method of intervention needs to be tailored according to individual characteristics [1]. Early intervention for people with frailty will provide many benefits for both, the individuals and the health care system. The population mean of BMI is rising not only in East Asia including Japan but also all over the world [15]. Therefore, frailty is an issue to be addressed in countries that are experiencing the double burden of malnutrition, and the control of BMI may be given high priority to decrease the prevalence of frailty.

## 5. Conclusions

This study revealed a U-shaped relationship between the prevalence of frailty and BMI in community-dwelling Japanese older adults. The results indicate that a healthy BMI may reduce the prevalence of frailty. Thus, the risk of frailty should be evaluated in underweight and overweight people.

## Figures and Tables

**Table 1:** Characteristics of participants in the whole cohort and subcohort ^a^.

	Whole Cohort (*n* = 7191) ^b^		Subcohort (*n* = 1169) ^c^	
Age (years) ^d^	73.4	(6.2)	72.7	(5.4)
Women (*n* (%)) ^e^	3788	(52.7)	585	(50.0)
BMI (kg/m^2^) ^d^	22.7	(3.5)	22.6	(2.8)
Alcohol drinker (*n* (%)) ^e^	4691	(65.2)	812	(69.5)
Current smoker (*n* (%)) ^e^	769	(10.7)	94	(8.0)
Living alone (*n* (%)) ^e^	813	(11.3)	123	(10.5)
HSE (*n* (%)) ^e^	2442	(34.0)	440	(37.6)
Education ≥13 y (*n* (%)) ^e^	1607	(22.3)	313	(26.8)
MVPA (*n* (%)) ^e^	3160	(43.9)	621	(53.1)
Denture use (*n* (%)) ^e^	4332	(60.2)	692	(59.2)
No medication (*n* (%)) ^e^	1591	(22.1)	240	(20.5)
Hypertension (*n* (%)) ^e^	2789	(38.8)	474	(40.5)
Stroke (*n* (%)) ^e^	269	(3.7)	33	(2.8)
Heart disease (*n* (%)) ^e^	914	(12.7)	131	(11.2)
Diabetes (*n* (%)) ^e^	763	(10.6)	112	(9.6)
Hyperlipidemia (*n* (%)) ^e^	723	(10.1)	139	(11.9)

BMI, body mass index; HSE, high socioeconomic status; MVPA, moderate to vigorous physical activity. ^a^ Missing values were imputed using multiple imputation. ^b^ Number of people with missing data: alcohol status, 108; smoking status, 132; physical activity, 402; family structure, 487; socioeconomic status, 236; education, 667; denture use, 83; and medication use, 392. ^c^ Number of people with missing data: alcohol status, 16; smoking status, 17; physical activity, 15; family structure, 70; socioeconomic status, 31; education, 115; denture use, 12; and medication use, 57. ^d^ Continuous variables are shown as means and standard deviations. ^e^ Categorical variables are shown as numbers of people and percentages.

**Table 2:** Reproducibility and validity of self-reported height, body weight, and BMI.

	Self-Reported ^a^				Measurement (M) ^a^		Mean Difference (95% CI) ^b^				Correlation ^c^	
	Baseline Survey (BS)		Additional Survey (AS)				BS and AS		BS and M		BS and AS	BS and M
Total (*n* = 1169)												
Height (cm)	157.8	(8.6)	157.7	(8.5)	156.9	(8.7)	−0.1	(−0.2 to 0.0)	−0.9	(−1.0 to −0.8)	0.970	0.970
Body weight (kg)	56.4	(9.6)	56.8	(9.8)	56.8	(10.0)	0.4	(0.2 to 0.6)	0.4	(0.3 to 0.5)	0.958	0.965
BMI (kg/m^2^)	22.6	(2.8)	22.8	(2.9)	23.0	(3.0)	0.2	(0.1 to 0.3)	0.4	(0.3 to 0.5)	0.898	0.915
Men (*n* = 584)												
Height (cm)	164.2	(5.9)	164.0	(6.1)	163.5	(6.0)	−0.2	(−0.4 to −0.1)	−0.7	(−0.9 to −0.5)	0.930	0.940
Body weight (kg)	61.8	(8.4)	62.3	(8.4)	62.4	(8.8)	0.5	(0.3 to 0.7)	0.6	(0.3 to 0.8)	0.958	0.952
BMI (kg/m^2^)	22.9	(2.6)	23.1	(2.6)	23.3	(2.8)	0.2	(0.1 to 0.3)	0.4	(0.3 to 0.5)	0.910	0.916
Women (*n* = 585)												
Height (cm)	151.4	(5.4)	151.4	(5.3)	150.4	(5.5)	0.0	(−0.1 to 0.1)	−1.0	(−1.1 to −0.8)	0.940	0.920
Body weight (kg)	51.0	(7.5)	51.3	(7.8)	51.3	(7.8)	0.3	(0.1 to 0.6)	0.3	(0.1 to 0.5)	0.915	0.947
BMI (kg/m^2^)	22.2	(3.0)	22.4	(3.1)	22.7	(3.1)	0.2	(0.1 to 0.3)	0.5	(0.3 to 0.6)	0.888	0.912
<75 years (*n* = 770)												
Height (cm)	158.6	(8.3)	158.4	(8.2)	157.8	(8.4)	−0.2	(−0.3 to −0.1)	−0.8	(−0.9 to −0.7)	0.980	0.983
Body weight (kg)	57.2	(9.6)	57.5	(9.8)	57.5	(10.0)	0.3	(0.1 to 0.5)	0.3	(0.2 to 0.5)	0.957	0.964
BMI (kg/m^2^)	22.6	(2.8)	22.8	(2.9)	23.0	(3.0)	0.2	(0.1 to 0.3)	0.4	(0.3 to 0.5)	0.908	0.928
≥75 years (*n* = 399)												
Height (cm)	156.2	(8.9)	156.2	(8.9)	155.2	(9.1)	0.0	(−0.3 to 0.2)	−1.0	(−1.3 to −0.7)	0.954	0.946
Body weight (kg)	54.9	(9.4)	55.4	(9.5)	55.4	(9.8)	0.5	(0.2 to 0.7)	0.5	(0.3 to 0.7)	0.958	0.967
BMI (kg/m^2^)	22.4	(2.8)	22.6	(2.9)	22.9	(3.0)	0.2	(0.1 to 0.3)	0.5	(0.4 to 0.6)	0.879	0.889

AS, additional survey; BMI, body mass index; BS, baseline survey; CI, confidence intervals; M, measurement. ^a^ Values are expressed as means and standard deviations. The baseline survey was conducted on 29 July 2011, and the additional survey on 14 February 2012. Actual measurements were taken between March and April 2012. ^b^ Values are expressed as terms of mean difference and 95% CI. ^c^ Pearson’s correlation coefficient and the intraclass correlation coefficient were used.

**Table 3:** Characteristics of participants by BMI category ^a^.

	BMI Categories (kg/m^2^)											
	<18.5 (*n* = 562)		18.5–19.9 (*n* = 787)		20.0–22.4 (*n* = 2195)		22.5–24.9 (*n* = 2218)		25.0–27.4 (*n* = 1006)		≥27.5 (*n* = 423)	
Age (years) ^b^	75.6	(6.8)	74.0	(6.7)	73.3	(6.2)	72.9	(5.8)	72.8	(5.9)	72.8	(5.8)
Women (*n* (%)) ^c^	388	(69.0)	465	(59.1)	1205	(54.9)	1012	(45.6)	469	(46.6)	249	(58.9)
BMI (kg/m^2^) ^b^	17.3	(1.0)	19.3	(1.5)	21.3	(1.6)	23.7	(1.9)	26.1	(2.3)	29.6	(2.2)
Alcohol drinker (*n* (%)) ^c^	304	(54.1)	484	(61.5)	1443	(65.7)	1504	(67.8)	685	(68.1)	271	(64.1)
Current smoker (*n* (%)) ^c^	71	(12.6)	85	(10.8)	252	(11.5)	239	(10.8)	90	(9.0)	32	(7.6)
Living alone (*n* (%)) ^c^	63	(11.2)	98	(12.5)	250	(11.4)	257	(11.6)	104	(10.3)	41	(9.7)
HSE (*n* (%)) ^c^	184	(32.7)	271	(34.4)	779	(35.5)	738	(33.3)	347	(34.5)	123	(29.1)
Education ≥13 y (*n* (%)) ^c^	104	(18.5)	181	(23.0)	467	(21.3)	535	(24.1)	225	(22.4)	95	(22.5)
MVPA (*n* (%)) ^c^	186	(33.1)	341	(43.3)	1002	(45.7)	1045	(47.1)	441	(43.8)	145	(34.3)
Denture use (*n* (%)) ^c^	370	(65.8)	484	(61.5)	1323	(60.3)	1311	(59.1)	600	(59.6)	244	(57.7)
No medication (*n* (%)) ^c^	138	(24.6)	210	(26.7)	529	(24.1)	477	(21.5)	182	(18.1)	55	(13.0)
Hypertension (*n* (%)) ^c^	126	(22.4)	227	(28.8)	774	(35.3)	934	(42.1)	507	(50.4)	221	(52.3)
Stroke (*n* (%)) ^c^	21	(3.7)	27	(3.4)	76	(3.5)	90	(4.1)	37	(3.7)	18	(4.3)
Heart disease (*n* (%)) ^c^	52	(9.3)	90	(11.4)	258	(11.8)	289	(13.0)	148	(14.7)	77	(18.2)
Diabetes (*n* (%)) ^c^	41	(7.3)	63	(8.0)	213	(9.7)	236	(10.6)	127	(12.6)	83	(19.6)
Hyperlipidemia (*n* (%)) ^c^	34	(6.1)	72	(9.2)	209	(9.5)	232	(10.5)	110	(10.9)	66	(15.6)

BMI, body mass index; HSE, high socioeconomic status; MVPA, moderate to vigorous physical activity. ^a^ Missing values were supplemented by multiple imputation for the following factors: alcohol status, *n* = 108; smoking status, *n* = 132; physical activity, *n* = 402; family structure, *n* = 487; socioeconomic status, *n* = 236; education, *n* = 667; denture use, *n* = 83; and medication use, *n* = 392. ^b^ Continuous variables are shown as means and standard deviations. ^c^ Categorical variables are shown as numbers of people and percentages.

**Table 4:** Odds ratios for BMI and prevalence rates of frailty defined by the FP model and the KCL, calculated using multivariate logistic regression ^a.^.

	BMI Categories (kg/m^2^)											
	<18.5 (*n* = 562)		18.5–19.9 (*n* = 787)		20.0–22.4 (*n* = 2195)		22.5–24.9 (*n* = 2218)		25.0–27.4 (*n* = 1006)		≥27.5 (*n* = 423)	
BMI (kg/m^2^)	17.3	(1.0)	19.3	(0.4)	21.3	(0.7)	23.6	(0.7)	26.0	(0.7)	29.5	(2.1)
FP model												
Case (*n* (%))	142	(25.3)	154	(19.6)	313	(14.3)	276	(12.4)	127	(12.6)	82	(19.4)
Crude model	2.34	(1.84 to 2.97)	1.69	(1.34 to 2.12)	1.15	(0.96 to 1.38)	1.00	(Ref)	0.98	(0.78 to 1.25)	1.66	(1.25 to 2.20)
Model 1 ^b^	2.07	(1.62 to 2.65)	1.63	(1.29 to 2.05)	1.13	(0.94 to 1.36)	1.00	(Ref)	1.01	(0.80 to 1.29)	1.73	(1.30 to 2.31)
Model 2 ^c^	2.04	(1.58 to 2.63)	1.69	(1.33 to 2.14)	1.16	(0.96 to 1.41)	1.00	(Ref)	1.00	(0.78 to 1.27)	1.54	(1.15 to 2.07)
KCL												
Case (*n* (%))	312	(55.5)	297	(37.7)	750	(34.2)	723	(32.6)	345	(34.3)	208	(49.2)
Crude model	2.58	(2.14 to 3.12)	1.25	(1.06 to 1.48)	1.07	(0.95 to 1.22)	1.00	(Ref)	1.08	(0.92 to 1.26)	2.00	(1.62 to 2.47)
Model 1 ^b^	2.08	(1.70 to 2.54)	1.13	(0.94 to 1.35)	1.01	(0.88 to 1.15)	1.00	(Ref)	1.10	(0.93 to 1.30)	2.13	(1.71 to 2.66)
Model 2 ^c^	2.04	(1.65 to 2.54)	1.16	(0.96 to 1.41)	1.03	(0.90 to 1.19)	1.00	(Ref)	1.07	(0.90 to 1.28)	1.86	(1.47 to 2.35)

BMI, body mass index; FP, Fried phenotype; KCL, Kihon Checklist; Ref, reference. ^a^ The prevalence of frailty defined by FP model and the KCL in the Kyoto–Kameoka Study were 15.2% (1094 people) and 36.6% (2635 people), respectively. BMI values are shown as means and standard deviations. The prevalence rates of frailty are shown as numbers of people and percentages. Statistical values regarding the relationship between BMI and the prevalence of frailty are shown as odds ratio and 95% confidence intervals. ^b^ Model 1 was adjusted for age, sex, and region. ^c^ Model 2 was adjusted for age, sex, region, smoking habit, alcohol consumption, education history, number of drugs taken, family composition, economic status, physical activity, presence/absence of dentures, and history of hypertension, stroke, heart disease, diabetes, and dyslipidemia.

## Supplementary Materials

The following are available online at https://www.mdpi.com/2077-0383/9/5/1367/s1. Table S1: Odds ratios for BMI and prevalence rates of frailty defined by FP model, calculated using age and sex stratified multivariate logistic regression, Supplementary Table S2: Odds ratios for BMI and prevalence rates of frailty defined by the KCL, calculated using age and sex stratified multivariate logistic regression.
