# Supplementary material for: A U-Shaped Relationship between the Prevalence of Frailty and Body Mass Index in Community-Dwelling Japanese Older Adults: The Kyoto–Kameoka Study
Source: J Clin Med. 2020 May 6;9(5):1367. doi: 10.3390/jcm9051367 (PMC7290950; doi:10.3390/jcm9051367)
Supplement: Supplementary file 1 [file jcm-09-01367-s001.pdf]

Table S1. Odds ratios for BMI and prevalence rates of frailty defined by FP model, calculated using age and sex stratified multivariate logistic regression [a]

	BMI categories (kg/m²)											
	<18.5		18.5–19.9		20.0–22.4		22.5–24.9		25.0–27.4		≥27.5	
Men, *n*	174		322		990		1206		537		174	
BMI (kg/m²)	17.4	(0.9)	19.3	(0.4)	21.3	(0.7)	23.6	(0.7)	26.0	(0.7)	29.4	(1.8)
Case (*n* (%))	41	(23.6)	69	(21.4)	152	(15.4)	143	(11.9)	58	(10.8)	26	(14.9)
Crude model	2.32	(1.55 to 3.48)	2.01	(1.44 to 2.81)	1.34	(1.03 to 1.73)	1.00	(Ref)	0.88	(0.63 to 1.23)	1.29	(0.81 to 2.07)
Model 1 [b]	1.86	(1.22 to 2.82)	1.87	(1.33 to 2.63)	1.29	(0.99 to 1.68)	1.00	(Ref)	0.90	(0.64 to 1.27)	1.27	(0.78 to 2.04)
Model 2 [c]	1.77	(1.14 to 4.27)	1.91	(1.33 to 2.72)	1.34	(1.03 to 1.76)	1.00	(Ref)	0.90	(0.64 to 1.28)	1.14	(0.70 to 1.86)
Women, *n*	388		465		1205		1012		469		249	
BMI (kg/m²)	17.2	(1.0)	19.3	(0.4)	21.3	(0.7)	23.6	(0.7)	26.0	(0.7)	29.7	(2.2)
Case (*n* (%))	101	(26.0)	85	(18.3)	161	(13.4)	133	(13.1)	69	(14.7)	56	(22.5)
Crude model	2.26	(1.67 to 3.06)	1.45	(1.06 to 1.98)	1.00	(0.77 to 1.29)	1.00	(Ref)	1.10	(0.79 to 1.53)	1.87	(1.30 to 2.69)
Model 1 [b]	2.13	(1.56 to 2.92)	1.43	(1.04 to 1.97)	1.00	(0.77 to 1.31)	1.00	(Ref)	1.11	(0.79 to 1.55)	2.06	(1.42 to 2.99)
Model 2 [c]	2.12	(1.53 to 2.92)	1.51	(1.09 to 2.09)	1.03	(0.79 to 1.35)	1.00	(Ref)	1.06	(0.76 to 1.50)	1.86	(1.27 to 2.72)
<75 years, *n*	273		473		1359		1449		654		284	
BMI (kg/m²)	17.4	(0.9)	19.3	(0.4)	21.3	(0.7)	23.6	(0.7)	26.0	(0.7)	29.5	(2.0)
Case (*n* (%))	52	(19.0)	73	(15.4)	136	(10.0)	152	(10.5)	80	(12.2)	45	(15.8)
Crude model	2.02	(1.40 to 2.90)	1.55	(1.13 to 2.13)	0.95	(0.73 to 1.23)	1.00	(Ref)	1.19	(0.88 to 1.61)	1.66	(1.15 to 2.41)
Model 1 [b]	2.00	(1.38 to 2.89)	1.56	(1.13 to 2.14)	0.96	(0.74 to 1.24)	1.00	(Ref)	1.19	(0.88 to 1.61)	1.64	(1.13 to 2.38)
Model 2 [c]	1.97	(1.35 to 2.89)	1.64	(1.18 to 2.27)	1.00	(0.77 to 1.30)	1.00	(Ref)	1.16	(0.85 to 1.59)	1.38	(0.94 to 2.03)
≥75 years, *n*	289		314		836		769		352		139	
BMI (kg/m²)	17.2	(1.0)	19.3	(0.4)	21.3	(0.7)	23.5	(0.7)	26.0	(0.7)	29.6	(2.2)
Case (*n* (%))	90	(31.1)	81	(25.8)	177	(21.2)	124	(16.1)	47	(13.4)	37	(26.6)
Crude model	2.24	(1.62 to 3.10)	1.76	(1.26 to 2.45)	1.34	(1.03 to 1.75)	1.00	(Ref)	0.74	(0.51 to 1.09)	1.75	(1.11 to 2.74)
Model 1 [b]	2.15	(1.54 to 3.01)	1.71	(1.22 to 2.41)	1.34	(1.02 to 1.76)	1.00	(Ref)	0.79	(0.54 to 1.17)	1.88	(1.19 to 2.96)
Model 2 [c]	2.12	(1.49 to 3.01)	1.76	(1.24 to 2.50)	1.36	(1.03 to 1.80)	1.00	(Ref)	0.78	(0.53 to 1.16)	1.81	(1.13 to 2.90)

BMI, body mass index; FP, Fried phenotype; Ref, reference. [a] The prevalence of frailty defined by FP model in the Kyoto-Kameoka Study were 14.4% (489 people) in men, 16.0% (605 people) in women, 12.0% (538 people) in <75 years old, and 20.6% (556 people) in ≥75 years old. BMI values are shown as means and standard deviations. The prevalence rates of frailty are shown as numbers of people and percentages. Statistical values regarding the relationship between BMI and the prevalence of frailty are shown as odds ratio and 95% confidence intervals. [b] Model 1 was adjusted for age, sex, and region. [c] Model 2 was adjusted for age, sex, region, smoking habit, alcohol consumption, education history, number of drugs taken, family composition, economic status, physical activity, presence/absence of dentures, and history of hypertension, stroke, heart disease, diabetes, and dyslipidemia.

Table S2. Odds ratios for BMI and prevalence rates of frailty defined by the KCL, calculated using age and sex stratified multivariate logistic regression [a]

	**BMI categories (kg/m²)**											
	**<18.5**		**18.5–19.9**		**20.0–22.4**		**22.5–24.9**		**25.0–27.4**		**≥27.5**	
Men, *n*	174		322		990		1206		537		174	
BMI (kg/m²)	17.4	(0.9)	19.3	(0.4)	21.3	(0.7)	23.6	(0.7)	26.0	(0.7)	29.4	(1.8)
Case (*n* (%))	110	(63.2)	137	(42.6)	320	(32.3)	358	(29.7)	170	(31.7)	75	(43.1)
Crude model	4.07	(2.92 to 5.67)	1.75	(1.36 to 2.26)	1.13	(0.94 to 1.36)	1.00	(Ref)	1.10	(0.88 to 1.37)	1.79	(1.30 to 2.48)
Model 1 [b]	3.05	(2.16 to 4.32)	1.56	(1.20 to 2.03)	1.06	(0.87 to 1.28)	1.00	(Ref)	1.12	(0.90 to 1.41)	1.79	(1.28 to 2.51)
Model 2 [c]	2.94	(2.02 to 4.27)	1.59	(1.20 to 2.11)	1.05	(0.86 to 1.29)	1.00	(Ref)	1.18	(0.92 to 1.50)	1.73	(1.21 to 2.47)
Women, *n*	388		465		1205		1012		469		249	
BMI (kg/m²)	17.2	(1.0)	19.3	(0.4)	21.3	(0.7)	23.6	(0.7)	26.0	(0.7)	29.7	(2.2)
Case (*n* (%))	202	(52.1)	160	(34.4)	430	(35.7)	365	(36.1)	175	(37.3)	133	(53.4)
Crude model	1.93	(1.52 to 2.44)	0.93	(0.74 to 1.17)	0.98	(0.83 to 1.17)	1.00	(Ref)	1.06	(0.84 to 1.32)	2.03	(1.54 to 2.69)
Model 1 [b]	1.71	(1.32 to 2.21)	0.87	(0.68 to 1.12)	0.96	(0.80 to 1.16)	1.00	(Ref)	1.05	(0.82 to 1.34)	2.38	(1.77 to 3.21)
Model 2 [c]	1.68	(1.28 to 2.20)	0.90	(0.69 to 1.17)	1.00	(0.82 to 1.21)	1.00	(Ref)	0.96	(0.74 to 1.24)	1.96	(1.43 to 2.69)
<75 years, *n*	273		473		1359		1449		654		284	
BMI (kg/m²)	17.4	(0.9)	19.3	(0.4)	21.3	(0.7)	23.6	(0.7)	26.0	(0.7)	29.5	(2.0)
Case (*n* (%))	106	(38.8)	120	(25.4)	310	(22.8)	350	(24.2)	171	(26.2)	120	(42.3)
Crude model	1.99	(1.52 to 2.61)	1.07	(0.84 to 1.36)	0.93	(0.78 to 1.11)	1.00	(Ref)	1.11	(0.90 to 1.37)	2.30	(1.76 to 2.99)
Model 1 [b]	2.03	(1.54 to 2.68)	1.08	(0.85 to 1.38)	0.95	(0.80 to 1.13)	1.00	(Ref)	1.12	(0.91 to 1.39)	2.30	(1.76 to 3.01)
Model 2 [c]	2.07	(1.54 to 2.78)	1.14	(0.89 to 1.48)	0.98	(0.81 to 1.18)	1.00	(Ref)	1.09	(0.87 to 1.37)	1.97	(1.48 to 2.62)
≥75 years, *n*	289		314		836		769		352		139	
BMI (kg/m²)	17.2	(1.0)	19.3	(0.4)	21.3	(0.7	23.5	(0.7)	26.0	(0.7)	29.6	(2.2)
Case (*n* (%))	206	(71.3)	177	(56.4)	440	(52.6)	373	(48.5)	174	(49.3)	88	(63.3)
Crude model	2.63	(1.97 to 3.53)	1.37	(1.05 to 1.79)	1.18	(0.97 to 1.44)	1.00	(Ref)	1.04	(0.81 to 1.34)	1.83	(1.26 to 2.66)
Model 1 [b]	2.19	(1.62 to 2.96)	1.21	(0.92 to 1.60)	1.09	(0.89 to 1.34)	1.00	(Ref)	1.05	(0.81 to 1.37)	1.85	(1.26 to 2.72)
Model 2 [c]	2.02	(1.46 to 2.79)	1.19	(0.89 to 1.59)	1.09	(0.88 to 1.36)	1.00	(Ref)	1.04	(0.78 to 1.37)	1.69	(1.12 to 2.55)

BMI, body mass index; KCL, Kihon Checklist; Ref, reference. [a] The prevalence of frailty defined by the KCL in the Kyoto-Kameoka Study were 34.4% (1170 people) in men, 38.7% (1465 people) in women, 26.2 % (1177 people) in <75 years old, and 54.0% (1458 people) in ≥75 years old. BMI values are shown as means and standard deviations. The prevalence rates of frailty are shown as numbers of people and percentages. Statistical values regarding the relationship between BMI and the prevalence of frailty are shown as odds ratio and 95% confidence intervals. [b] Model 1 was adjusted for age, sex, and region. [c] Model 2 was adjusted for age, sex, region, smoking habit, alcohol consumption, education history, number of drugs taken, family composition, economic status, physical activity, presence/absence of dentures, and history of hypertension, stroke, heart disease, diabetes, and dyslipidemia.
